# BAG-1 expression and function in human cancer

**DOI:** 10.1038/sj.bjc.6600538

**Published:** 2002-10-07

**Authors:** R I Cutress, P A Townsend, M Brimmell, A C Bateman, A Hague, G Packham

**Affiliations:** Cancer Research UK Oncology Unit, Cancer Sciences Division, Mail Point 824, School of Medicine, University of Southampton, Southampton General Hospital, Southampton SO16 6YD, UK; Department of Pathology, Southampton General Hospital, Tremona Road, Southampton SO16 6YD, UK; Department of Oral and Dental Science, University of Bristol, Bristol Dental School, Lower Maudlin Street, Bristol BS1 2LY, UK

**Keywords:** BAG-1, heat shock, apoptosis, transcription, cancer

## Abstract

BAG-1 is a multifunctional protein that interacts with a wide range of target molecules to regulate apoptosis, proliferation, transcription, metastasis and motility. Interaction with chaperone molecules may mediate many of the effects of BAG-1. The pathways regulated by BAG-1 play key roles in the development and progression of cancer and determining response to therapy, and there has been considerable interest in determining the clinical significance of BAG-1 expression in malignant cells. There is an emerging picture that BAG-1 expression is frequently altered in a range of human cancers relative to normal cells and a recent report suggests the exciting possibility that BAG-1 expression may have clinical utility as a prognostic marker in early breast cancer. However, other studies of BAG-1 expression in breast cancer and other cancer types have yielded differing results. It is important to view these findings in the context of current knowledge of BAG-1 expression and function. This review summarises recent progress in understanding the clinical significance of BAG-1 expression in cancer in light of our understanding of BAG-1 function.

*British Journal of Cancer* (2002) **87**, 834–839. doi:10.1038/sj.bjc.6600538 www.bjcancer.com

© 2002 Cancer Research UK

## BAG-1 STRUCTURE

**Figure 1:**
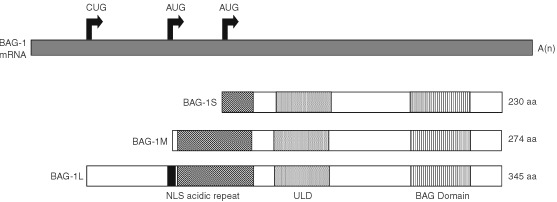
Schematic diagram of the human BAG-1 isoforms. The BAG-1 mRNA and the position of the alternate translation initiation sites that give rise to the three human BAG-1 isoforms is shown at the top. The domain structure of the BAG-1 isoforms is shown underneath (NLS, nuclear localization sequence; ULD, ubiquitin-like domain).

BAG-1 proteins are expressed as multiple isoforms generated by alternate translation initiation from a single mRNA (Packham *et al*, 1997; Takayama *et al*, 1998; Yang *et al*, 1998). The major human BAG-1 isoform, BAG-1S, initiates at an internal AUG codon, whereas translation of the larger BAG-1L and BAG-1M proteins initiate at upstream CUG and AUG codons, respectively. The proteins therefore share a common C-terminus and the larger isoforms have additional N-terminal sequences (Figure 1

).

Various domains have been identified within BAG-1 proteins. We initially identified a potential nuclear localisation signal (NLS) within the unique N-terminal domain of BAG-1L, consistent with the predominantly nuclear localisation of this isoform (Packham *et al*, 1997; Takayama *et al*, 1998; Yang *et al*, 1998; Brimmell *et al*, 1999). By contrast, BAG-1S and BAG-1M lack this sequence and BAG-1S is largely located in the cytoplasm whilst BAG-1M partitions between the nucleus and cytoplasm (Packham *et al*, 1997; Takayama *et al*, 1998; Yang *et al*, 1998). The nuclear/cytoplasmic distribution of BAG-1 isoforms is regulated under some conditions and it is important to recognise that nuclear BAG-1 immunostaining in cancer cells may not be a reliable measure of BAG-1L expression. For example, BAG-1M relocates from the cytoplasm to the nucleus after heat shock and in response to hormonal stimulation (Schneikert *et al*, 1999; Zeiner *et al*, 1999). Thus, nuclear BAG-1 expression may indicate either high levels of BAG-1L or relocalisation of BAG-1S or BAG-1M to the nucleus in response to specific signals in the tumour microenvironment.

All BAG-1 isoforms contain a C-terminal ‘BAG domain’ which plays a key role in mediating many BAG-1 functions (Figure 1). This domain of approximately 100 amino-acid residues defines a family of related BAG proteins conserved throughout phylogeny, of which there are at least six in the human (Takayama and Reed, 2001; Sondermann *et al*, 2001). The core of the BAG domain comprises two anti-parallel alpha-helices that mediate interaction with the HSC70 and HSP70 heat shock proteins (Briknarova *et al*, 2001; Sondermann *et al*, 2001), molecular chaperones that bind proteins in non-native states assisting them to reach functional confirmations. HSC70 and HSP70 proteins comprise a peptide-binding domain that interacts with denatured polypeptides and a regulatory ATPase domain. BAG-1 interacts with the ATPase domain, leaving the peptide-binding domain available for further interactions with protein substrates. BAG-1 regulates the chaperone function of HSC70 and HSP70 (Hohfeld, 1998) and mutation of specific amino-acid residues important for binding to chaperone proteins abrogates at least some BAG-1 functions (Briknarova *et al*, 2001).

The BAG-1 C-terminus also mediates interaction with the serine/threonine kinase Raf-1, which is normally activated by RAS to stimulate the mitogen-activated protein (MAP) kinase signalling cascade. This signalling pathway is important for proliferation and survival, and BAG-1 activates Raf-1 independent of RAS (Song *et al*, 2001). Thus, BAG-1 overexpression provides a potential mechanism by which tumours lacking oncogenic RAS mutations might activate MAP kinase pathway mediated proliferative and survival signals. Raf-1 and HSP70 interact at partially overlapping sites and therefore their binding to BAG-1 is competitive.

**Figure 2:**
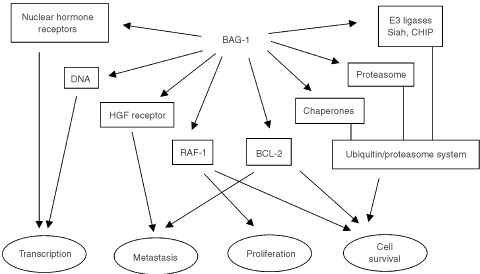
BAG-1 binding partners and functions. BAG-1 interaction partners are indicated. Some of these interactions are direct, whereas others are probably mediated via binding to chaperone molecules, e.g., NHR. Interactions of BAG-1 with chaperones, E3 ligases and the proteasome suggest a key role in regulating the ubiquitin/proteasomal degradation system. Biological activities ascribed to BAG-1 are indicated below along with some potential molecular targets that might contribute to these effects. However, it is important to note that definitive evidence linking specific BAG-1 target molecules to biological responses is often lacking.

In addition to these direct binding partners, several other proteins have been reported to interact with BAG-1 (Figure 2

), including nuclear hormone receptors (NHR), the anti-apoptotic Bcl-2 protein and some tyrosine kinase receptors such as the hepatocyte growth factor and platelet derived growth factor receptors (for reviews see Cato and Mink, 2001; Takayama and Reed, 2001). Although definitive proof is often lacking, it is possible that much of this binding is indirect and mediated via the peptide binding activities of HSC70/HSP70 (Hohfeld, 1998).

All BAG-1 isoforms contain a ubiquitin-like domain (ULD), similar to ubiquitin and ubiquitin-like proteins that appears to be essential for at least some biological effects (Luders *et al*, 2000; Hohfeld *et al*, 2001; Takayama and Reed, 2001). Ubiquitin is a small protein that when covalently attached to target proteins via the action of ubiquitin E3 ligases serves to target proteins for ATP-dependent degradation via the proteasome, the major non-lysosomal proteolytic complex. Although the precise function of the ULD in BAG-1 is unknown, BAG-1 isoforms are very stable proteins suggesting that they are not generally targets for degradation by the ubiquitin/proteasome system (Luders *et al*, 2000) and are not covalently attached to other proteins. Other ULD-containing proteins are important for regulating ubiquitylation/proteolysis and consistent with this, BAG-1 interacts with the E3 ligases CHIP and Siah-1 and stimulates CHIP-mediated ubiquitylation of substrates such as Raf-1 (Demand *et al*, 2001). BAG-1 interacts with the proteasome. The amino-terminus of BAG-1 is required for this interaction, and one potential mechanism of action of BAG-1 is by linking chaperone molecules with the proteasome (Luders *et al*, 2000; Hohfeld *et al*, 2001).

BAG-1 proteins contain different numbers of copies of repeats rich in acidic amino-acids. Human BAG-1L and BAG-1M contain 10 copies whereas BAG-1S contains four (Packham *et al*, 1997). The function of the repeats remains unclear since this part of the molecule is not essential for suppression of apoptosis, but is required for regulating glucocorticoid receptor (GR)-dependent transcription (Schneikert *et al*, 1999).

## BAG-1 FUNCTION

Overexpression of BAG-1 suppresses activation of caspases and apoptosis induced by a very broad range of agents in different cell types (Hohfeld, 1998; Takayama and Reed, 2001), for example chemotherapeutic agents, radiation and growth factor withdrawal. Therefore, in addition to contributing to reduced cell death in cancer development, BAG-1 may also contribute to resistance to important therapeutic modalities. The finding that BAG-1 can independently associate with Raf-1 or Bcl-2 provides at least two potential mechanisms by which BAG-1 promotes survival (Song *et al*, 2001). Heat shock proteins are also required for cell survival and direct activation of these functions may also be important. Suppression of apoptosis might contribute to the ability of BAG-1 to promote metastatic spread (Takaoka *et al*, 1997; Yawata *et al*, 1998). Alternatively, increased metastasis may be mediated by enhanced cell motility (Yawata *et al*, 1998).

A second key function of BAG-1 of likely importance for cancer is regulation of NHR. BAG-1 potentiates activity of oestrogen receptors (ER) (R Cutress, PA Townsend and G Packham, unpublished data), which mediates proliferative, and survival responses to oestrogens in hormone dependent breast cancers and is the target for anti-hormone therapies such as tamoxifen. The androgen receptor (AR) is important in prostate cancer and BAG-1 increases sensitivity of AR expressing cells to androgens and decreases sensitivity to cyproterone acetate, an anti-androgen used clinically in the treatment of prostate cancer (Froesch *et al*, 1998). BAG-1 also modulates the activity of the vitamin D3 receptor (VDR) and represses the activity of the retinoic acid receptor (RAR), thyroid receptor (TR) and glucocorticoid receptor (GR) (Cato and Mink, 2001; Takayama and Reed, 2001). The effects of BAG-1 on some nuclear hormone receptors requires the BAG-1 carboxy-terminus and is likely to involve chaperone molecules which are known to be important for NHR function. This has been demonstrated most clearly for the AR where point mutations which ablate HSC70 binding prevent BAG-1 regulation (Briknarova *et al*, 2001). Effects on NHR may be partly through BAG-1 triggered conformational changes mediated by the heat shock proteins which may be important for altering hormone binding capacity or affinity. This is not the case for all NHR however, as for example the HSC70 binding region within the carboxy-terminus of BAG-1 is not required for binding to the RAR (Cato and Mink, 2001). Biochemical and mutagenic analyses therefore suggest that there may not be a single mechanism to account for BAG-1's modulation of nuclear hormone receptor activity (Cato and Mink, 2001).

Although all BAG-1 isoforms interact with chaperone molecules and possess anti-apoptotic activity, regulation of NHR is frequently specific for the BAG-1L and/or BAG-1M proteins. For example, only BAG-1L regulates the AR (Froesch *et al*, 1998) and ER (R Cutress, PA Townsend and G Packham, unpublished data) whereas both BAG-1L and BAG-1M regulate the GR. It is not clear whether the frequent specificity for the larger BAG-1 isoforms stems from the nuclear localisation of these proteins or from a requirement for additional HSC/HSP70 independent functions encoded by the amino-termini of these isoforms. For example, the acidic-rich repeat has been suggested to be important for conferring GR-regulating activity on BAG-1L and M (Schneikert *et al*, 1999). Alternately, non-specific DNA binding mediated by the basic amino-acid residues of the N-terminal NLS and/or transactivating activity of the larger BAG-1 proteins might play a role in stabilising receptor : DNA complexes and/or recruiting cooperating transcription factors (Zeiner *et al*, 1999).

We are some way from a complete understanding of how BAG-1 exerts its effects on such a diverse array of biological pathways, including proliferation, apoptosis and metastasis in addition to its effects on transcription. As with NHR, many BAG-1 functions are thought to be dependent on interaction with the HSC70 and/or HSP70 chaperone molecules (Hohfeld, 1998). Despite this, simple regulation of the refolding action of these proteins by BAG-1 is not sufficient for all the observed actions of BAG-1, since other BAG-1 protein regions are required for biological effects in addition to the BAG domain that binds HSC70/HSP70. BAG-1 may act as a scaffold molecule, to functionally link chaperone function with specific target molecules (Hohfeld *et al*, 2001; Takayama and Reed, 2001). For example, BAG-1 may protect cells from the apoptotic effects of cell stress induced by radiation or heat shock by enhancing the delivery of chaperone-bound denatured proteins to the proteasomal degradation system.

BAG-1 is the prototypical member of a family of BAG domain containing proteins, which bind to and regulate chaperone molecules. Although BAG-6 also contains a ULD, other BAG family proteins contain distinct protein : protein interaction motifs, for example BAG-3 contains a WW domain and PXXP motifs in addition to its BAG domain. Similar to the proposed role of BAG-1 in linking chaperones and the proteasome, recruitment of chaperone molecules to other molecular targets via specific BAG proteins may represent a common theme of cell growth control, conserved through evolution (Takayama and Reed, 2001).

Mechanisms of action of BAG-1 that do not occur through direct HSC70/HSP70 binding, for example, the activation of Raf-1 by BAG-1 are also likely to be important (Song *et al*, 2001). The binding of HSP70 and Raf-1 for BAG-1 is competitive and the high levels of HSP70 that accumulate in stressed cells may displace Raf-1, shutting down important signals for survival and proliferation. Therefore, BAG-1 isoforms may act as a ‘molecular switch’ in signalling pathways, that direct cells towards different states ‘depending on whether environmental conditions are hospitable or stressful’ (Song *et al*, 2001; Takayama and Reed, 2001).

## BAG-1 AND BREAST CANCER

Given the impact of BAG-1 overexpression on multiple growth control pathways, there has been considerable interest in studying the significance of BAG-1 in human cancer. Four large immunohistochemical studies of the expression and clinical significance of BAG-1 in breast cancer have been reported and these are summarised in Table 1

**Table 1:**
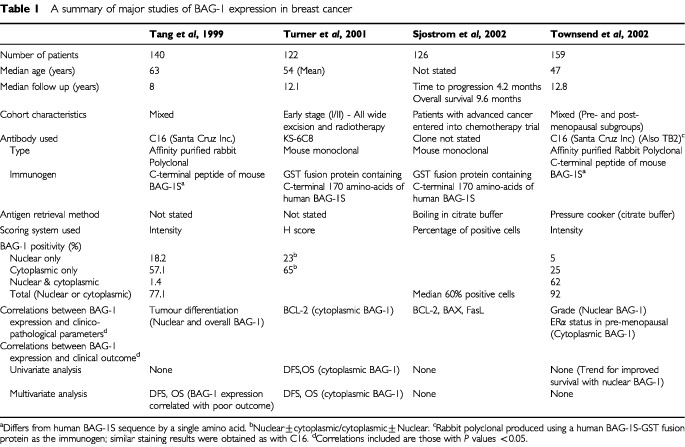
A summary of major studies of BAG-1 expression in breast cancer

(Tang *et al*, 1999; Turner *et al*, 2001; Sjostrom *et al*, 2002; Townsend *et al*, 2002). Some of the results from these studies are inconsistent and further studies are required to fully understand the role of BAG-1 expression in breast cancer (Cutress *et al*, 2001). A consistent finding is that relatively high levels of cytoplasmic BAG-1 expression are detected in two thirds or more cases of breast cancer. Changes in BAG-1 expression can be detected in benign lesions such as sclerosing adenosis, and in ductal carcinoma *in situ* suggesting that they might represent a relatively early event in breast cancer development (Brimmell *et al*, 1999). By contrast, the extent of nuclear BAG-1 expression differs widely in these studies, possibly for reasons discussed below, ranging from as low as 20% to almost 70%. Moreover, the proportion of tumours with both nuclear and cytoplasmic expression varies widely from just 1% to more than 60%.

A strong relationship between nuclear BAG-1 expression and tumour grade/differentiation has been identified in two studies, with relatively high levels of nuclear BAG-1 expression in low grade tumours (Tang *et al*, 1999; Townsend *et al*, 2002). By contrast, Turner *et al* (2001) reported no correlation between tumour grade and BAG-1 expression. Correlations with other clinical markers, such as oestrogen receptor alpha (ERα) and BCL-2 expression have been reported in various studies, but these appear to be not as strong as the correlation with grade and are more variably detected (Table 1).

Turner *et al* (2001) reported an overall 10-year survival for women with early stage breast cancer of 82% with high cytoplasmic BAG-1 levels *vs* 42% survival with low BAG-1 levels. Cytoplasmic BAG-1 status predicted outcome in both univariate and multivariate analyses, and also retained predictive value in a subset of their patients with pathologically negative lymph nodes. This is particularly interesting since it was suggested that this might provide a means by which node negative patients with a relatively poorer prognosis could be selected for further adjuvant therapies, and conversely enable better prognosis node negative patients to avoid such therapies with their concomitant side effects. This exciting finding awaits confirmation (Tang *et al*, 1999; Townsend *et al*, 2002). Tang *et al* (1999) found no correlations between BAG-1 expression and outcome in univariate analysis and conversely reported that increased BAG-1 expression correlated with decreased disease free and overall survival in a multivariate analysis controlled for tumour differentiation. In our own study no correlations were found between cytoplasmic BAG-1 and outcome (Townsend *et al*, 2002). We did, however, observe a tendency for patients with nuclear BAG-1 expression to have slightly (but not statistically significantly) improved outcomes, consistent with the correlation we found with low tumour grade.

The ability of BAG-1 expression to predict response to adjuvant therapy has currently only been assessed in one study. Sjostrom *et al* (2002) found that BAG-1 expression did not predict time to progression or overall survival in patients with advanced breast cancer entered into a randomised controlled trial comparing docetaxel with sequential methotrexate and 5-fluorouracil. BAG-1 status therefore appears not to predict response to chemotherapy, but, unlike other studies demonstrating prognostic significance, BAG-1 subcellular localisation was not analysed. Further work is required to assess the ability of BAG-1 to predict response to adjuvant therapy, in particular given the role of BAG-1 in modulation of NHR function, and the importance of adjuvant hormonal therapy in breast cancer.

## BAG-1 AND OTHER MALIGNANCIES

BAG-1 expression has also been studied in a range of other cancer types (Table 2

**Table 2:**
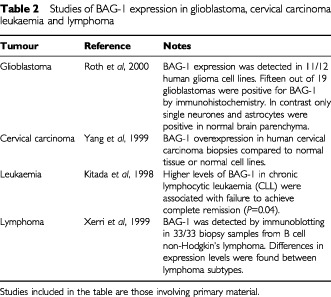
Studies of BAG-1 expression in glioblastoma, cervical carcinoma leukaemia and lymphoma

). Rorke *et al* (2001) have studied the expression of BAG-1 by immunohistochemistry in non-small cell lung cancer. Consistent with studies in other tumour types they found that approximately two thirds of tumours expressed high levels of BAG-1. In particular they found no correlations between BAG-1 and clinico-pathological parameters, but did find that, similar to the study of Turner *et al* (2001) in breast cancer, cytoplasmic expression of BAG-1 independently correlated with improved overall survival.

Several groups have studied expression of BAG-1 in human squamous cell carcinomas. Yamauchi *et al* (2001) found that, in contrast to breast and lung cancer, nuclear expression of BAG-1 in laryngeal tumours conferred a worse disease-free survival after radiotherapy. In oral squamous cell carcinomas, Shindoh *et al* (2000) demonstrated increased BAG-1 expression in tumour tissue relative to adjacent normal epithelium in 60–80% of samples. By contrast, our study of 64 oral squamous cell carcinomas and 17 samples of normal oral epithelium, revealed reduced nuclear BAG-1 expression in oral squamous cell carcinomas (*P*=0.036) compared to normal oral epithelium (Hague *et al*, 2002).

BAG-1 enhances metastasis in experimental models (Takaoka *et al*, 1997; Yawata *et al*, 1998) and Shindoh *et al* (2000) demonstrated that BAG-1 was expressed in 89% of primary tumours with nodal metastasis compared to 38% of tumours without. BAG-1 expression levels were determined by densitometry and although this alleviates the subjectivity of scoring BAG-1 labelling intensity to some extent it does not give information on whether the immunoreactivity is nuclear or cytoplasmic. We examined BAG-1 expression by immunohistochemistry in paired samples of primary tumour and matched lymph node metastasis and demonstrated statistically significant increased cytoplasmic expression in 8 of the 13 metastatic tumours relative to the corresponding primary tumour (*P*=0.021). However, in contrast to the results of Shindoh *et al* (2000), no significant difference in BAG-1 expression was detected in the primary tumours between patients with and without associated lymph node metastases. We determined the predominant BAG-1 immunostaining intensity and it is possible that the maximal intensity may provide improved prognostic value for metastatic potential. Alternatively, the environment at the site of the metastasis may induce BAG-1 expression. The presence or absence of lymph node metastases are of strong prognostic significance and evaluation of these events in relation to prognosis in larger cohorts is warranted.

## CONCLUSIONS

Consistent with its effects on a diverse range of important cell growth control pathways, there is increasing evidence that BAG-1 expression is frequently altered in human cancer. Studies have focused on breast cancer, non-small cell lung cancer and squamous cell carcinoma of the head and neck, and there are indications that BAG-1 expression may also be altered in glioblastoma, cervical cancer and haemopoietic malignancies (Table 2). Although some inconsistencies have been reported, there appears to be broad agreement that BAG-1 is overexpressed in breast and non-small cell lung cancer and that this can correlate with clinical parameters and improved patient outcome. Further work, including prospective trials, are required to confirm the exciting possibility that BAG-1 expression might be used as a prognostic marker in early breast cancer (Turner *et al*, 2001). Larger prospective studies should be more representative of the spectrum of breast cancer as a whole and have increased power to detect independent prognostic predictors in multivariate analysis, in particular in the presence of possible confounding associations such as tumour grade and ERα status.

As discussed by Turner *et al* (2001) it is not immediately apparent why an anti-apoptotic protein is associated with good prognosis. This kind of observation is not unique, however, since expression of Bcl-2 is also associated with good prognosis in breast cancer, for example. Multiple alterations contribute to carcinogenesis, and tumours that counter apoptosis by overexpressing Bcl-2 and/or BAG-1 might represent one class of tumours. Other tumours may have disabled apoptotic responses through accumulation of distinct changes (e.g., p53 mutation, ErbB2 overexpression) which might have more profound effects on cell death sensitivity or additionally affect proliferative pathways resulting in more aggressive tumour growth. Effects of BAG-1 on NHR function in hormone sensitive tumours may also play a role.

Some of the inconsistencies reported will undoubtedly stem from experimental differences and the subjective nature of immunohistochemical analyses. The signal detected in an immunohistological assay is not linear with the antibody concentration, nor with many other of the technical parameters (Wynford-Thomas, 1992). For example in the four breast cancer studies discussed here, various antibodies, antigen retrieval methods and scoring systems (intensity *vs* ‘H-score’) were used, all of which might have significantly influenced the results (Table 1). A change in antigen retrieval technique or scoring threshold might alter the proportion of tumours that are considered to be positive for expression and might contribute to the variation in detection of nuclear BAG-1 expression in breast cancer. Such difficulties, amongst others, have been encountered with the immunohistochemical analysis of p53 (Wynford-Thomas, 1992).

Although BAG-1 is part of a family of related proteins, we have seen no evidence of cross reaction of these antibodies with other BAG family members, but this remains a theoretical possibility, especially in antibodies raised against antigens that contain the conserved BAG domain.

Another important difference between studies is the composition of the patient cohorts, which are likely to differ in many ways e.g., menopausal status and stage and treatment. Since all studies to date are retrospective, the patient selection and exclusion criteria, treatment protocols and outcome measures are both different between studies and not necessarily available in their entirety. Since BAG-1 can impact on multiple cell control pathways, the impact of specific patterns of BAG-1 expression on survival may depend greatly on the treatment applied in different cohorts. For example, only BAG-1L regulates ERα function (R Cutress, PA Townsend and G Packham, unpublished data) and the expression of nuclear BAG-1L might be particularly important in determining response to hormonal therapy in breast cancer. By contrast, all BAG-1 isoforms appear to possess anti-apoptotic activity and therefore cytoplasmic BAG-1S might be particularly important in determining responses to chemotherapy. Therefore, the significance of nuclear or cytoplasmic staining might differ depending on the treatment modality. Unfortunately, there is great heterogeneity between previous studies as to treatment, and the differences between these studies must be interpreted in the light of this.

It is also likely that the key targets of BAG-1 will differ between cell types. For example, ERα is a key regulator of breast epithelial cells and BAG-1L may therefore be a key determinant of survival in breast cancer. By contrast, in oral cancer, suppression of retinoid-induced differentiation may be crucial. Since the BAG-1S isoform suppresses RAR function, overexpression of BAG-1S might also be of importance. Thus, we should not expect a simple pattern of changes in BAG-1 expression in all cancer types due to differences in ‘key’ BAG-1 targets in different cell types.

A major factor that complicates the interpretation of immunohistochemical analyses stems from the fact that there are multiple BAG-1 isoforms that can be functionally distinct. The consequences of high level BAG-1L expression might be very different from those of relocalisation of cytoplasmic isoforms to the nucleus. Unfortunately, these proteins are not discriminated by the antibodies currently used in immunohistochemical analyses of cancer samples. Since the same mRNA encodes all BAG-1 isoforms it is also not possible to use RNA-based approaches to measure their relative levels of expression. BAG-1L-specific antibodies will be useful in analysing the expression of specific BAG-1 isoforms in tumour samples.

Further complexity is added by consideration of issues of function. It is not known for example if mutations occur within BAG-1, and if so if these might affect expression levels and what the functional consequences might be. BAG-1 may be phosphorylated under some conditions (Cato and Mink, 2001), but the functional significance of this and other potential post-translational modifications (such as BAG-1 ubiquitylation (Sourisseau *et al*, 2001)) are poorly understood and presumably not discriminated by immunohistochemistry with current antibodies.

It is becoming increasingly clear that expression of BAG-1 is frequently altered in human cancer and it is possible that expression analysis might be of clinical utility. Further studies, including prospective trials, are required and, given the ability of BAG-1 to target multiple biological pathways, these should be sufficiently large to allow pre-defined subgroup analysis to determine the effect of BAG-1 on survival in patients treated with specific therapeutic regimens (e.g., hormonal therapy *vs* chemotherapy). Despite the interesting and encouraging results obtained from studies of BAG-1 in breast cancer, the ability of BAG-1 overexpression to promote tumour formation in transgenic animals is yet to be tested. Such studies may produce compelling evidence that BAG-1 plays a causal role in cancer development. The development and application of isoform specific antibodies will also be key to characterise the significance of specific BAG-1 isoforms. This should enable dissection of the exact relationship between differing BAG-1 isoforms and clinical outcome, and to further clarify the possible role of BAG-1 as a novel prognostic marker and therapeutic target in a wide range of malignancies.
